# Energy Efficiency Analysis: Biomass-to-Wheel Efficiency Related with Biofuels Production, Fuel Distribution, and Powertrain Systems

**DOI:** 10.1371/journal.pone.0022113

**Published:** 2011-07-13

**Authors:** Wei-Dong Huang, Y-H Percival Zhang

**Affiliations:** 1 Biological Systems Engineering Department, Virginia Tech, Blacksburg, Virginia, United States of America; 2 Environmental Division, College of Earth and Space Science, University of Science and Technology of China, Hefei, China; 3 Institute for Critical Technology and Applied Science (ICTAS), Virginia Tech, Blacksburg, Virginia, United States of America; 4 DOE BioEnergy Science Center (BESC), Oak Ridge, Tennessee, United States of America; 5 Gate Fuels Inc, Blacksburg, Virginia, United States of America; Université Joseph Fourier, France

## Abstract

**Background:**

Energy efficiency analysis for different biomass-utilization scenarios would help make more informed decisions for developing future biomass-based transportation systems. Diverse biofuels produced from biomass include cellulosic ethanol, butanol, fatty acid ethyl esters, methane, hydrogen, methanol, dimethyether, Fischer-Tropsch diesel, and bioelectricity; the respective powertrain systems include internal combustion engine (ICE) vehicles, hybrid electric vehicles based on gasoline or diesel ICEs, hydrogen fuel cell vehicles, sugar fuel cell vehicles (SFCV), and battery electric vehicles (BEV).

**Methodology/Principal Findings:**

We conducted a simple, straightforward, and transparent biomass-to-wheel (BTW) analysis including three separate conversion elements -- biomass-to-fuel conversion, fuel transport and distribution, and respective powertrain systems. BTW efficiency is a ratio of the kinetic energy of an automobile's wheels to the chemical energy of delivered biomass just before entering biorefineries. Up to 13 scenarios were analyzed and compared to a base line case – corn ethanol/ICE. This analysis suggests that BEV, whose electricity is generated from stationary fuel cells, and SFCV, based on a hydrogen fuel cell vehicle with an on-board sugar-to-hydrogen bioreformer, would have the highest BTW efficiencies, nearly four times that of ethanol-ICE.

**Significance:**

In the long term, a small fraction of the annual US biomass (e.g., 7.1%, or 700 million tons of biomass) would be sufficient to meet 100% of light-duty passenger vehicle fuel needs (i.e., 150 billion gallons of gasoline/ethanol per year), through up to four-fold enhanced BTW efficiencies by using SFCV or BEV. SFCV would have several advantages over BEV: much higher energy storage densities, faster refilling rates, better safety, and less environmental burdens.

## Introduction

The sustainability revolution from non-renewable sources to renewable sources is the defining challenge of our time [1], [2], [3]. Mobility usually represents the level of a civilization [4], [5]. Light-duty passenger vehicles, which constitute the largest type of transportation energy consumption among different transportation modes, have some special requirements, such as high energy storage capacity in a small container (e.g., ∼50 liters), high power output (e.g., ∼20–100 kW per vehicle), affordable fuel (e.g., $∼20–30/GJ), affordable vehicle, low costs for rebuilding the relevant infrastructure, fast charging or refilling of the fuel (e.g. several min per time), and safety concerns [5], [6], [7]. Such strict requirements result in limited choices for fuels and respective powertrain systems. Here powertrain refers to the group of components that generate power from stored energy and deliver it to wheels of vehicles running on the road surface, including the engine, transmission, drive shaft, differentials, and wheels [8], [9]. Therefore, current light-duty passenger vehicles mainly rely on non-renewable liquid fuels and internal combustion engines (ICE). But the depletion of crude oil, accumulation of greenhouse gases, concerns of national energy security, and creation of manufacturing jobs are motivating the development of sustainable transportation biofuels based on local renewable biomass [1], [3], [9], [10].

Most ethanol is made from corn kernels and sugarcane, but this practice raises heated debate due to competition with food supplies; furthermore, its contribution to the transport sector is minimal or modest [1], [11]. Lignocellulosic biomass is presently believed to be the only major renewable bioresource that can produce a significant fraction of liquid transportation fuels and renewable materials in the future [2], [9], [11], [12] because the overall energy stored in phytobiomass each year is approximately 30-fold of the energy consumed for transportation [9], [13]. But the future role of biomass in the transport sector remains in debate [1], [14], [15].

A great variety of biofuels can be produced from lignocellulose biomass, including cellulosic ethanol [10], [16], butanol and/or long chain alcohols [17], [18], electricity [19], [20], bioalkanes [21], fatty acid esters [6], [22], [23], hydrogen [24], [25], [26], [27], hydrocarbons [28], [29], and waxes [22]. The biofuels that will become short-, middle- and long-term transportation fuels is a matter of vigorous debate. Among them, some biofuels may have a particular niche market. For example, jet planes require high-density liquid fuels [6], [17], [21], [22]. First, the analysis presented here is restricted to the largest transportation fuel market – fuels for light-duty passenger vehicles. Second, this analysis starts from less costly lignocellulosic biomass that can be collected and delivered at reasonable costs (e.g., ∼$60–100 dollars per ton) [9], [11]. Third, algal biofuel production or other renewable electricity generation (e.g., solar and wind electricity) is not covered in this paper.

Several types of powertrain systems have been developed to convert stored energy to kinetic energy, including internal combustion engines (e.g., gas ICE, diesel ICE, jet turbine, and rocket turbine), external combustion engines (e.g., steam engine and steam turbine), and electric motors. Because of special requirements of passenger vehicles, such as weight-to-power ratio (e.g., one to several g/W), engine costs (e.g., tens dollars/kW), and engine lifetime (e.g., ∼5,000 h), only three engines are acceptable for passenger vehicles: gas ICE, diesel ICE, and electric motor. Considering electricity stored in batteries and possible on-board electricity generation systems (e.g., hydrogen proton exchange membrane (PEM) fuel cell) plus their hybrids, this analysis attempted to compare six current and future powertrain systems: gas-based ICE vehicles (ICE-gas) [7], [8], hybrid electric vehicles based on gasoline ICE (HEV-gas) [30], hybrid electric vehicles based on diesel (HEV-diesel) [30], fuel cell vehicles based on compressed H_2_ (FCV) [31], [32], [33], [34], battery electric vehicles (BEV) [20], [32], and sugar (hydrogen) fuel cell vehicles (SFCV) [3], [5], [9].

Numerous life cycle analyses (LCA) have been conducted to investigate the potential impacts of biomass/biofuels on energy applications, greenhouse gas emissions, and even water footprint [10], [14], [15], [35], [36], [37], [38], [39], [40], [41], [42], [43], [44]. But such analyses rely heavily on numerous assumptions, uncertain inputs (e.g., fertilizers, pesticides, farm machinery), energy conversion coefficients among different energy forms and sources, system boundaries, and so on. For example, conflicting conclusions have been made even for well-known corn ethanol biorefineries [10], [36], [37].

**Figure 1:**
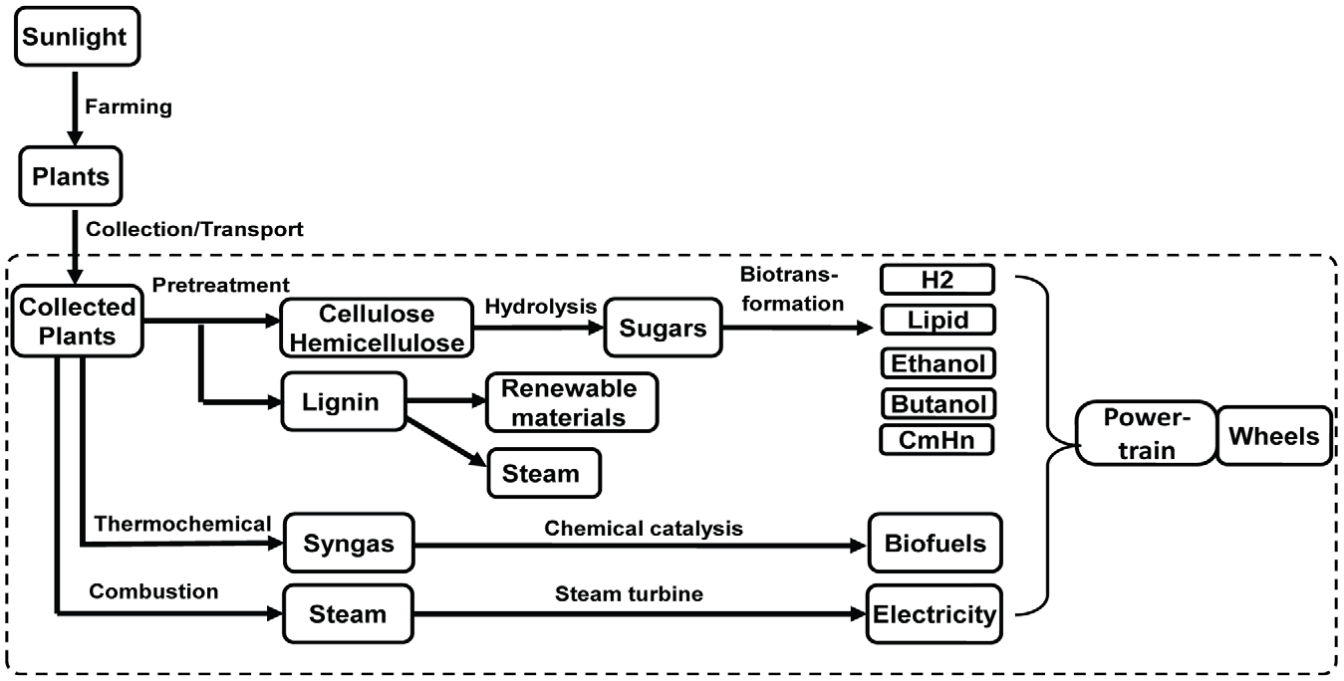
Different pathways for biofuels production from lignocellulosic biomass. The current energy efficiency analysis focuses on the delivered biomass-to-wheel efficiency related with conversion, transportation and power train systems.

Here we suggest developing an energy efficiency analysis for biomass-to-wheel (BTW), a ratio of kinetic energy of the wheels of an automobile to the chemical energy of delivered biomass (Fig. 1). Conducting this BTW analysis is simple and straightforward because it not only avoids uncertainties or debates for (i) biomass production-related issues, (ii) feedstock collection and transport, and (iii) land use change, but also excludes water consumption issues and greenhouse gas emissions in the whole biosystem. Therefore, energy efficiency analysis (but not life cycle analysis) may not only be helpful in narrowing down numerous choices before more complicated LCA and techno-economic analyses are conducted, but may also increase the transparency of such analyses.

**Figure 2:**
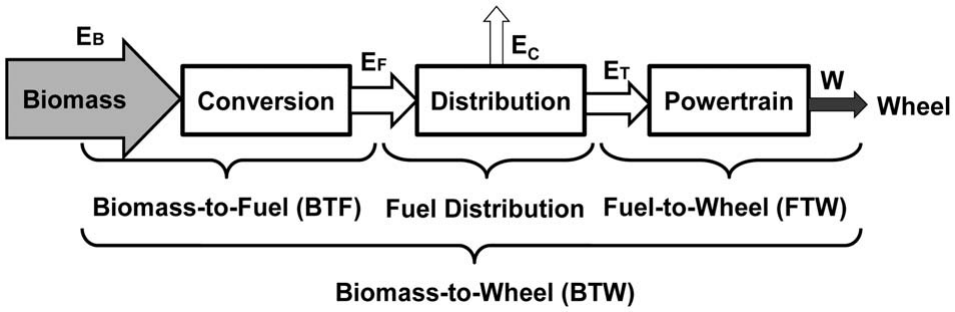
The scheme of energy efficiency analysis for biomass-to-wheel efficiency calculation -- 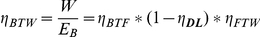.

In this article, we present a simple biomass-to-wheel (BTW) efficiency (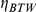) analysis methodology involving three elements -- biomass-to-fuel (BTF), fuel distribution, and fuel-to-wheel (FTW) (Fig. 2). Using this method, 13 combinations of different biomass-to-biofuel approaches and their respective powertrain systems were analyzed as compared to a baseline – corn-ethanol-ICE. The identification of high BTW efficiency scenarios would help make a more informed decision for how to utilize (limited) biomass resource more efficiently. Following this, a more detailed LCA should be conducted for evaluating potential impacts associated with identified inputs and releases and for compiling an inventory of more relevant energy and material inputs as well as environmental effects.

## Methods

The biomass-to-wheel efficiency (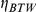), an energy conversion ratio of an automobile's kinetic energy to the harvested and delivered biomass in the front of the door of biorefineries, involves three sequential elements – biomass-to-fuel production, fuel transport and distribution, and the powertrain system responsible for the fuel-to-wheel conversion (Fig. 2). The BTW efficiency is the lumped efficiency from chemical energy in biomass to kinetic energy for vehicle driving. The 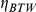 value can be calculated as below
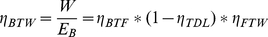
(1)where

W is the kinetic energy transferred to wheels;


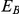
 is the chemical combustion energy of the biomass, where dry corn stover as a typical biomass contains ∼65% carbohydrates (cellulose and hemicellulose, mainly), ∼18% lignin, ∼5% ash, ∼12% other organic molecules [45], [46]; and the 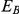 value is 16.5 MJ of low heating value/kg of corn stover [47];


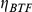
is the biomass-to-fuel (BTF) efficiency through biorefineries or power stations without significant inputs or outputs of other energy;


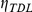
 is the fuel loss efficiency during its transport and distribution; and


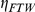
 is the fuel-to-wheel (FTW) efficiency from the fuel to kinetic energy through powertrain.

The 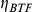 value can be calculated as below
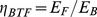
(2)where E_F_ is the fuel produced in biorefineries or power stations. The 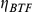 values of current corn ethanol as a reference range from 46% to 50% [48], and the value of 49% is chosen as a baseline [10]. Through the biomass sugars platform, potential biofuels include cellulosic ethanol, butanol, fatty acid esters (ester-diesel), hydrogen, and methane. Through syngas made by a thermochemical pathway, potential biofuels are ethanol, hydrogen, methanol, dimethyl ether (DME), FT-diesel, and electricity [49], [50], [51]. Also, electricity can be produced through direct combustion for the generation of steam followed by a steam turbine/generator, or biomass integrated gasification combined cycle (BIGCC) to fuel cells (Table 1).

**Table 1:** Biomass-to-fuel (BTF) efficiency through different biomass utilization pathways.

Biofuel	Technology	Feedstock	Efficiency	Original Data	Original Data unit	Reference
corn ethanol	fermentation	corn	46.4%	0.372	L/kg dry	[95]
	fermentation	corn	49.4%	0.396	L/kg dry	[10]
	fermentation	corn	50.1%	0.402	L/kg dry	[48]
cellulosic ethanol	fermentation	corn stover	48.4%	0.298	kg/kg	[45]
	fermentation	corn stover	55.6%	0.342	kg/kg	[53]
sugar	hydrolysis	corn stover	55.8%	0.652	kg/kg	[53]
	hydrolysis	corn stover	61.1%	0.714	kg/kg	[58]
hydrogen	gasification	wood	55.0%	55.00	%LHV	[57]
	gasification	almond shells	70.8%	74%	HHV	[58]
methanol	gasification	wood	50.9%	0.477	kg/kg	[59]
	gasification	lignocellulose	54.9%	59.0	%HHV	[58]
DME	gasification	energy crop	39.0%	39–56.8%	LHV	[60]
FT-diesel	gasification	lignocellulose	41.4%	42.0	%HHV	[31]
	gasification	lignocellulose	52.0%	52.0	%LHV	[61]
ester micro-diesel	fermentation	glucose	7.2%	14.0	% theoretical efficiency	[22]
	fermentation	glucose	36.5%	64	%LHV	[6]
butanol	fermentation	glucose	46.7%	0.350	g/g glucose	[17]
	fermentation	glucose	52.8%	92.6%	LHV	[6]
methane	fermentation	ley crops	62.2%	10.6	GJ/dry ton	[54]
	fermentation	energy maize	81.3%	0.374	m^3^/kg dry maize	[55]
electricity	boiler	lignocellulose	25–43%	25–43%	LHV	[62]
electricity	BIGCC	lignocellulose	45.0%	45.0%	LHV	[63]
	BIGCC	lignocellulose	32–40%	32–40%	LHV	[62]
electricity	molten carbonate FC	lignocellulose	40.2%	40.2%	LHV	[64]
electricity	FC	lignocellulose	51.0%	51.0%	LHV	[65]

Different powertrains are required to convert different biofuels to the kinetic energy of the wheels. The 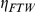 value can be calculated as a ratio between the kinetic energy on wheels (W) and fuel energy in the tank (E_T_):
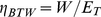
(3)


For liquid biofuels, powertrain systems are gasoline ICE, HEV-gas, and HEV-diesel. Fuel cell vehicles run on stored compressed hydrogen, through a PEM fuel cell stack and an electric motor. The sugar fuel cell vehicle (SFCV) is a hypothetical powertrain system, where sugar is a hydrogen carrier, an on-board bioreformer generates high-purity hydrogen for PEM fuel cell stacks, and the remaining powertrain parts are the same as FCV [5], [9]. The battery electricity vehicle (BEV) is a battery/motor system based on rechargeable batteries that can store electricity.

The 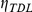 value can be calculated as fuel consumed for its transport and distribution from biorefineries to end-users (vehicles)
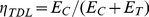
(4)where E_C_ is the energy consumed in the process of fuel transport and distribution, E_T_ is the fuel energy delivered to end users (i.e., powertrains), and E_F_  =  E_C_ + E_T._

Fuel losses during transport and distribution were obtained from the Argonne National Laboratory's model Greet 1.8c [52]. Detailed data sources and efficiency calculations are available in Table 2.

**Table 2 pone-0022113-t002:** Distribution energy efficiency loss*.

Distribution energy efficiency loss		Input data (Greet 1.8c *)	
Biofuel	Efficiency loss %	Energy input	Unit
Electricity	8.00	8.00	%
FT-diesel	1.53	15,557	btu/mmbtu
Dimethylester	3.10	31,980	btu/mmbtu
Methanol	3.29	34,021	btu/mmbtu
Hydrogen	17.5	211,654	btu/mmbtu
Methane	7.54	81,550	btu/mmbtu
Sugar	1.47	5,979	btu/bushel
ester-diesel	0.75	7,541	btu/mmbtu
Butanol	1.35	13,636	btu/mmbtu
Ethanol	1.71	17,387	btu/mmbtu

*http://www.transportation.anl.gov/modeling_simulation/GREET/index.html.

## Results

**Figure 3:**
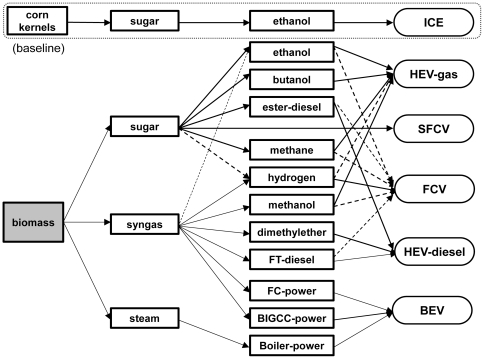
Scenarios of the production of fuels from biomass and their respective fuel power train systems. Solid lines represent the scenarios that we analyzed; the dotted lines represent possible scenarios that we did not analyze.

Different scenarios of fuel production through sugar, syngas, and steam platforms as well as six different powertrains viz. internal combustion engine vehicle (ICE), hybrid electric vehicle-gas (HEV-gas), hybrid electric vehicle-diesel (HEV-diesel), (hydrogen) fuel cell vehicle (FCV), battery electric vehicle (BEV), and sugar fuel cell vehicle (SFCV) are shown in Figure 3.

### Biomass-to-fuel efficiency (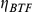)

**Figure 4:**
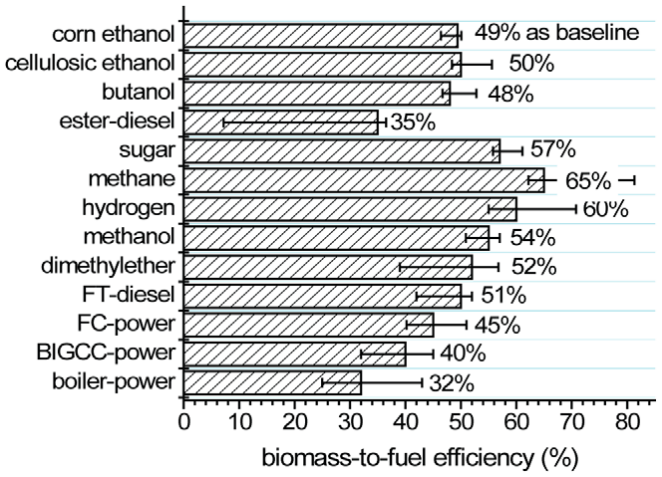
Comparison of biomass-to-fuel (BTF) efficiency in the biorefineries or power stations.

All biomass-to-fuel efficiency data plus their original data and units for different biomass pathways are listed in Table 1, and their representative 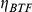 values are presented in Fig. 4.

In this study, we use corn stover as a representative biomass, in which total carbohydrates (including cellulose and hemicellulose) account for approximately 60–65% of combustion energy in biomass. Through the biochemical (sugar) pathway, the remaining chemical energy in biomass, mainly lignin, is consumed for running pretreatment as well as sugar isolation and product separation [45]. In general, ∼35–40% of the chemical energy of biomass is enough to run biorefineries without external energy input [45], [53]. The 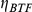 values for sugar-to-biofuels mainly depend on sugar isolation yields and sugar-to-fuel yields during microbial fermentation or enzymatic biotransformation. In this study, the 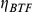 value is 57%, i.e., ∼88–95% of sugar release from biomass, in agreement with data elsewhere [45]. Given sugar yields of 88–99% for cellulose and hemicellulose and sugar-to-ethanol yields of 92–95%, the 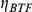 value of cellulosic ethanol would be 50%, with a range of 48–56% [10], [53]. Given the sugar-to-butanol yields from 82% (now) [17] to 93% (future) [6], the 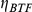 value for butanol fermentation would be about 48% with a range of 47–53%. Methane can be produced by anaerobic fermentation mediated by a microbial consortium, where microorganisms convert all organic components except non-hydrolytic lignin to methane. Therefore, 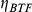 values range from 62 to 81% [54], [55]. The practical 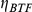 value of methane may be approximately 65%, higher than 50% (ethanol) and 48% (butanol). In contrast to anaerobic biofuels fermentations, long chain fatty acid esters (microdiesel) must be produced from sugars through semi-aerobic fermentation due to an imbalance of NAD(P)H [6], [22], [23]. Because semi-aerobic fermentation consumes a significant amount of sugar for the synthesis of cell mass than anaerobic fermentation, less carbohydrate would be allocated to the production of microdiesel [6], [56]. The 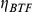 values of the ester-diesel fermentation would be about 35%, in the range of 7 to 37% depending on the fuel yields, from 13% [22] to 64% (future) [6].

Syngas can be produced from biomass through gasification – partial combustion at temperatures above 1000 K and in the presence of oxygen and/or water. Gasification is a relatively mature technology, so a significant fraction of biomass must be consumed for partial combustion, resulting in relatively low energy efficiencies, even though all organic components can be utilized [49], [50], [51]. The 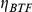 values for hydrogen generation from biomass range from 55% [57] to 71% [58] with a mean value of ∼60%. The 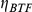 values for methanol, DME and FT-diesel vary from 51% [59] to 55% [31], from 39% to 57% [60], and from 41% [31] to 52% [61], respectively. Preferred 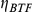 values are 54% (methanol), 52% (DME), and 51% (FT-diesel), respectively. Clearly, the 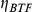 values for liquid biofuels (methanol, DME and FT-diesel) are lower than those of hydrogen because of more catalysis steps and their accompanied energy losses.

Bioelectricity can be produced simply through boiler/steam turbine technology, with 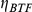 values ranging from 25% (now) to 43% (future) [62]. The assumed 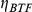 value is approximately 32%. Biomass integrated gasification, combining gas and steam turbine for electricity production (BIGCC), would have improved overall efficiencies, ranging from 32 to 45% [62], [63]. In order to increase electricity generation efficiency without restriction of the second law of thermodynamics for turbines, the integrated biomass gasification and fuel cells would have 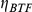 values of 40 to 51% [64], [65].

### Transport and distribution loss efficiency (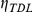)

**Figure 5:**
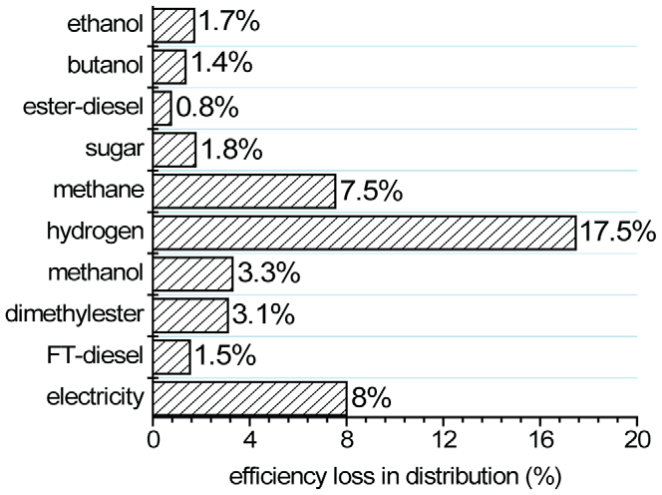
Comparison of transport and distribution loss efficiency for different fuels.

Fuel distribution processes consume a fraction of fuel produced from biorefineries or power stations (Fig. 5). Original data and units were obtained from the Greet1.8c software (Table 2). Typical 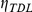 values for different fuels after normalization are shown in Figure 5. In general, liquid biofuels have similar efficiency losses (e.g., 0.8–3.3%). Gaseous fuels, such as hydrogen and methane, have more energy consumption for their compression, transport, refilling, and so on. The 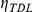 values are 17% for compressed hydrogen and 8% for compressed methane (Greet1.8c). The well-documented distribution efficiency of electricity is 92%, i.e., 8% of electricity is lost during its distribution (Greet1.8c).

### Fuel-to-wheel efficiency (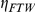)

Two major internal combustion engines for passenger vehicles are gasoline Otto (spark plug firing) ICE and diesel (compression ignition) ICE. Gasoline ICEs have a low weight-to-power ratio (e.g., ∼1 g engine per W output) but their maximum efficiencies are relatively low, approximately 32%, due to low compression ratios [66]. In contrast, diesel ICEs have a higher weight-to-power ratio (e.g., ∼3–4 g engine per W output) and a much higher energy conversion efficiency, more than 40% [66]. It is reasonable that diesel ICEs are widely used in heavy-duty trucks, tanks, and tractors. In Europe, diesel ICE passenger vehicles are more popular mainly due to higher fuel costs and more climate change concerns. Audi A3 vehicles based on ICE-diesel have 35.4 miles per gallon of diesel, higher than ICE-gasoline (24.7 miles per gallon of gasoline) [67], suggesting a ∼26% enhancement in 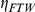 efficiency. (Note: the volumetric energy density of diesel is ∼13–14% higher than that of gasoline) [7].

Practical 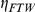 values of ICEs are much lower than their maximum efficiency because of (i) the engines operate at ∼70% of their maximum efficiency during most driving conditions, (ii) ∼17% loss for engine idling, (iii) ∼2% consumption for accessories (e.g., air conditioning, lighting), and (iv) ∼25% loss in transmission [30], [66], [68]. Therefore, the 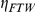 for ethanol-ICE is approximately 14% as a baseline [69], and this value would be improved through higher compression rate ethanol engine and better transmission [70], [71], [72]. Advanced diesel vehicles are expected to have 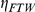 values of 20–24% [71]; the 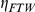 value of 23% is used in this study.

Hybrid electric vehicles (HEV) can eliminate idling losses, allow a small engine to work at nearly optimal conditions, and utilize braking energy with regenerative braking [30], [73]. Therefore, advanced HEV-gas is estimated to have 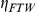 values of 29–34% [30], [74]. Similarly, the 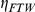 values of HEV-diesel can be increased to 32–38%, with a preferred value of 37%.

The hydrogen fuel cell vehicle (FCV) is a complicated powertrain system involving compressed hydrogen, FEM fuel cells, an electric motor, and a rechargeable battery [32], [75]. FCVs feature zero tailpipe pollution and high energy conversion efficiencies due to PEM fuel cells, whose theoretical energy efficiency from hydrogen to electricity is up to 83%. As a result, many companies have attempted big research FCV projects, and some of them produced prototype FCVs, such as the GM Sequel, the BMW Hydrogen 7, the Ford Focus FCV-Fuel Cell, the Toyota Fine X, and the Honda FCX Clarity. The 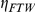 values of FCVs range from 41 to 54% [32], [75], with a mean value of 45%. SFCVs based on FCVs would have an on-board bioreformer that can convert the sugar slurry to high-purity hydrogen and absorb waste heat from PEM fuel cells. Because the efficiency of sugar-to-hydrogen is 107% based on low heating value [9], [24], [25], the 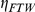 value for SFCV is estimated to be 48% with a range of 44–57%.

**Figure 6:**
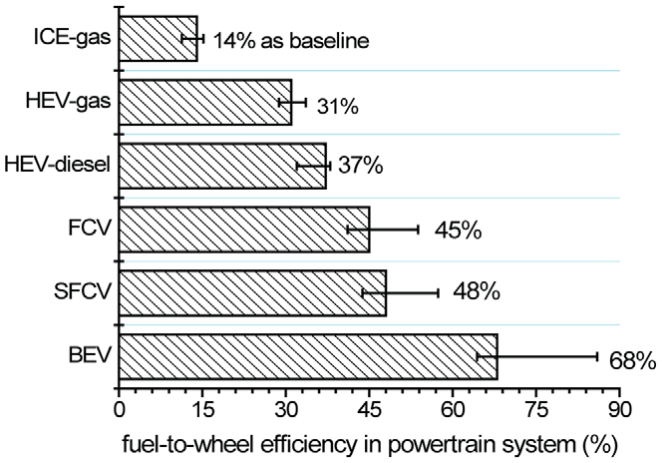
Comparison of fuel-to-wheel (FTW) efficiency for different powertrain systems.

Battery electric vehicles (BEV) have the highest 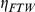
values, although they still have some energy losses in battery recharging and release, storage loss, motor, and so on [32], [76]. BEVs have predicted 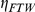 values from 64 to 86% [32], [76], [77], with a mean value of 68%. All fuel-to-wheel efficiencies of different vehicles are summed up in Table 3 and Fig. 6.

**Table 3:** Fuel-to-wheel (FTW) efficiency for different powertrains.

Powertrain	Efficiency	Reference
ICE-gas	11.3–15.2%	[30], [69], [70], [71]
ICE-diesel	20–24%	[71]
HEV-gas	28.8–31.4%	[30], [74]
HEV-diesel	34.6–37.6%	based on HEV-gas [30], [74] and ICE-diesel [71]
FCV	41.0–53.8%	[32], [75]
SFCV	43.7–57.3%	based on FCV plus sugar to H_2_ biotransforming efficiency [6], [24], [25]
BEV	64.4–86%	[32], [76], [77]

### Biomass-to-Wheel (BTW) efficiency (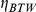)

**Figure 7:**
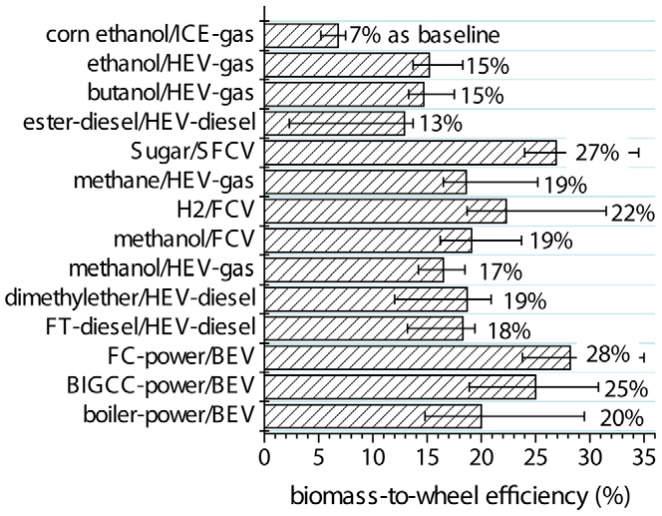
Comparison of biomass-to-wheel (BTW) efficiency for different biomass utilization scenarios.

A combination of 12 kinds of biofuel production approaches and 6 kinds of advanced powertrains for passenger vehicles results in more than 20 scenarios (Fig. 3). In this analysis, 14 scenarios were calculated (Fig. 7). The current corn ethanol/ICE scenario has 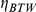 value of ∼7%, i.e., only 7% of the chemical energy in corn kernels is converted to the kinetic energy on wheels, implying a great potential in increasing biomass utilization efficiency. An ethanol HEV-gas system would double 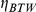 values to 14–18%, suggesting the importance of developing hybrid electric vehicles based on available liquid fuel distribution system. There is no significant difference in 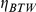 between butanol and ethanol, but butanol may have other important future applications, such as powering jet planes. The 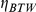 values of methane/HEV-gas and methanol/HEV-gas are 19% and 17%, respectively, higher than those of ethanol and butanol, mainly due to higher product yields. Since ICE-diesel has higher 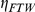 efficiencies than ICE-gas, the scenarios based on HEV-diesel through DME and FT-diesel (except ester-diesel) would have higher 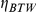 values than HEV-gas scenarios. For ester-diesel, a significant amount of energy is lost during aerobic fermentation due to thermodynamic and bioenergetic limits [6], resulting in low 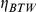 values. Even for the niche jet fuels market, the production of ester-diesel through semi-aerobic microbial fermentation might not be competitive with anaerobic butanol fermentation [78] and a high-energy-retaining efficiency hybrid of biocatalysis and chemical catalysis [28].

Although (hydrogen) fuel cell vehicles (FCVs) have higher 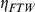 efficiencies than ICE-gas and ICE-diesel, the H_2_/FCV scenario shows ∼46% and ∼15% 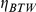 enhancements over ethanol HEV-gas and DME HEV-diesel, respectively, because significant energy loss in hydrogen distribution discounts FCV's advantages over HEV-diesel. The sugar/SFCV scenario would have very high 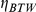 values of approximately 27% due to lower energy consumption in fuel transport and heat recapture in the sugar-to-hydrogen biotransformation, compared to the H_2_/FCV scenario.

BEV scenarios are among the highest 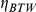 values, from 20% to 28%, with increasing electricity generation efficiencies from direct combustion, BIGCC, to FC-power.

## Discussion

Conducting energy efficiency analysis is simpler, faster, and less controversial than conducting life cycle analysis because the latter heavily depends on so many different assumptions and uncertain inputs. Here we present a straightforward energy efficiency analysis from biomass to wheels for different options, which contains three elements. Each element can be analyzed separately and adjusted individually; most of which have data well-documented in literature (Tables 1–3). Because of the same input and output in all cases, an increase in energy conversion efficiency nearly equals impact reductions in carbon and water footprints on the environment. Most of the results obtained from this biomass-to-wheel analysis were in good agreement with previous, more complicated life cycle analyses, supporting the validity of this methodology. Our analysis suggested that the hydrogen fuel cell vehicle (H_2_/FCV) scenario would have at least comparable efficiency with or a little higher than hybrid electric vehicle (HEV) systems, which was supported by a previous paper [76]. Another analysis suggested that the H_2_/fuel cell scenario had three times higher efficiency than ethanol/internal combustion engines (ICE) [33], in good agreement with our analysis (Fig. 7). Through comparison of four biofuels (i.e., hydrogen, methanol, Fischer–Tropsch (FT)-diesel, and ethanol) and two powertrain systems (i.e., ICE and FCV), they recommended FCV due to the highest energy efficiency [31]. These data were comparable with our analysis (Fig. 7). Both the sugar/sugar fuel cell vehicle (SFCV) and fuel cell (FC)-power/battery electric vehicle (BEV) scenarios would have nearly four times that of corn ethanol/ICE-gas, implying the importance of enhancing BTW efficiency in each conversion element.

### A new solution -- sugar-fuel cell vehicles (SFCV)

The concept of SFCV was proposed to address problems associated with H_2_/FCV, such as high-density hydrogen storage in FCV, low-cost sustainable hydrogen production, costly hydrogen distribution infrastructure, and safety concern [9], [25]. In this system, renewable sugar (carbohydrate) is suggested as a high hydrogen density carrier, with a gravimetric density of 8.33% mass H_2_ and a volumetric density of more than 100 g H_2_ per liter [3], [5], [9]. Transportation and distribution of the sugar/water slurry or sugar slurry would be easily achieved using available infrastructure. This hypothetical SFCV based on FCV would contain a sugar tank and an on-board sugar-to-hydrogen bioreformer, with a combined sugar tank and bioreformer volume that is much smaller than a compressed hydrogen tank or other hydrogen storage approaches [3], [5]. The sugar/water slurry would be refilled rapidly into the sugar container in SFCVs at local sugar stations; the on-board biotransformer would convert the sugar solution to high-purity hydrogen and carbon dioxide using a stabilized enzyme cocktail; and a small-size hydrogen storage container would serve as a buffer, balancing hydrogen production and consumption. In addition, feeding a mixture of CO_2_/H_2_ or pure hydrogen in the proton exchange membrane (PEM) fuel cells would dramatically decrease system complexity and greatly increase system operation performance, and the waste heat release from PEM fuel cells would be coupled to the heat needed by the bioreformer. Electrical energy from PEM fuel cells would be sent to the motor controller/motor/gears to generate kinetic energy [9]. When extra kinetic energy is needed for acceleration or start-up, electrical energy stored in the rechargeable battery would be released, like in a hybrid electric vehicle [9]. The on-board bioreformer in SFCVs, mediated by the thermoenzyme cocktails under modest reaction conditions (e.g., ∼80°C and ∼1 atm), may be capable of providing high-purity hydrogen at a rate of ∼23.5 g H_2_/L/h or higher. Given a bioreformer size of 42.8 L, one kg of hydrogen per hour could then be produced to drive the PEM fuel cell stack, followed by the electric motor [5]. High-speed biohydrogen production rates have been implemented by high cell-density microbial fermentation [79]. It is widely known that enzymatic reactions usually are at least one order-of-magnitude faster than microbial fermentations because the former has no cellular membrane to slow down mass transfer and much higher biocatalyst loadings, without the dilution of other biomacromolecules (e.g., DNA, RNA, other cellular proteins) [3], [56], [80], [81]. Current gasoline/ICE cars require maintenance every 3,000 miles (e.g., 4,800 km) or 3 months, i.e., 50–100 driving hours. Discovery of thermophilic enzymes that are stable at ∼80°C for more than 100 h has been demonstrated, for example, *T. maritima* 6-phosphogluconate dehydrogenase [82]. We expect that enzyme deactivation in the biotransformer will be solved through infrequent service maintenance, similar to the oil/air filter change for gasoline/ICE vehicles. Several technical obstacles of SFCVs include poor enzyme stability, labile and costly coenzymes, low reaction rates, and complicated system configuration and control [3], [9], [56], [80]. A huge potential market (e.g., nearly one trillion of US dollars per year) provides the motivation to solve these issues within a short time. Current progress includes the discovery of thermostable enzymes from extremophiles and low-cost production of recombinant enzymes [80], [82], [83], [84], [85], [86], engineering redox enzymes that can work on small-size biomimetic cofactors [56], [87], [88], and accelerating hydrogen generation rates [5], [9], [24], [89].

### SFCV is better than BEV

Although the biomass-to-wheel efficiency may be the most important criterion in analyzing future transportation systems, many factors were related with future choices, including energy storage density, system compactness, fuel costs, infrastructure, safety, operation reliability, environmental costs, resource availability, technology maturity, and improvements potential. Because the energy densities of lithium ion batteries (0.46–0.72 MJ/kg) [90], [91] are much lower than those of liquid fuels (∼30–40 MJ combustion energy/kg) and sugars (∼11–14 MJ electricity/kg sugar) [3], [5], BEVs will have a very short driving distance, making the BEV poorly suited for long-distance transportation [32]. If the energy densities of rechargeable batteries were increased by 10-fold in the future, safety concerns would likely come into play, slowing or even preventing wide deployment of such batteries in BEVs. In fact, it is impossible to increase energy densities of lithium rechargeable batteries by 10-fold due to physical limits [90]. Metal/air batteries are supposed to have the highest energy storage density of all batteries [90]. But regeneration of oxidized metals is so energy intensive that metal/air batteries may be too costly for the transport sector. SFCV would have a comparable 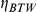 with the FC-boiler/BEV scenario but with much longer driving distances based on the same fuel weight (i.e., broader applications). Also, refilling of solid sugar or sugar/water slurry into SFCVs would be much faster and safer than recharging batteries for BEVs or refilling compressed hydrogen for FCVs. If the obstacles to ultra-fast recharging and the life-time of batteries were solved, a huge infrastructure investment would be required for upgrading electrical grids, sockets for quick recharging, power stations, etc. Since SFCV would have ∼3.4 times the FTW efficiency of ethanol/ICE-gas (Fig. 6), one kg of sugar (i.e., 17 MG/kg) would release more kinetic energy than one kg of gasoline (i.e., 46.4 MJ/kg) from ICE-gas. Thus, the mass of sugar delivered in the future may be less than the mass delivered by the current liquid gasoline/diesel distribution system. Another advantage is the much shorter sugar slurry transportation distance compared to that of gasoline/diesel, due to local production and distribution. The distribution of sugar would be done based on available goods distribution systems. Since SFCVs use biodegradable enzymes as catalysts, they would greatly decrease the environmental burdens related to BEVs, such as disposing and recycling used batteries.

### Beyond BTW

Assessment of any energy system is really challenging because it involves so many factors. Generally speaking, efficiency and cost are usually the two most important criteria. Since thermodynamics (energy efficiency) determine economics in the long term, SFCVs and FC-power/BEV seemed to be long-term winner candidates, but SFCVs have other important advantages. Currently and in the short term, costs mostly determine market acceptance and dominance. But cost analysis is more complicated than energy efficiency analysis, because the former involves direct costs (e.g., fuel, vehicle, etc.), indirect costs (e.g., vehicle service, taxes, subsidies, infrastructure costs for repairing and rebuilding, resource availability, etc.), and hidden costs (e.g., safety, toxicity, waste treatment, greenhouse gas emissions, military expenditures, etc.). In the short term, cellulosic ethanol plus HEV-gas and methane-HEV-gas may be the most promising options.

### Potential roles of biomass

It was important to estimate the role of US biomass resources in the future transport sector. The net primary production of biomass in the USA would be approximately 9.83 billion of dry metric tons in 2030, based on the current net primary (biomass) production with an annual growth rate of 1% [92], mainly due to higher photosynthesis yields accompanied with rising CO_2_ levels [93], [94]. Considering the fact that gasoline/bioethanol consumption in 2008 was approximately 140 billion gallons per year and an assumed annual growth rate of 1%, a switch from ethanol/ICE to sugar/SFCV would require net biomass energy of 11.60 EJ/year in 2030. That is, approximately 700 million metric tons of biomass in 2030, i.e., ∼7.1% of calculated annual US biomass (i.e., net primary production including natural ecosystems plus agricultural systems), would be sufficient to meet 100% of transportation fuel needs for light-duty passenger vehicles.

On the prospect of meeting transportation energy needs at acceptable fuel costs, we would like to suggest that short-term or middle-term solutions would be ethanol/butanol/methane plus HEV considering available current fuel distribution infrastructure and enhanced BTW efficiencies. In the long term, SFCVs will likely win over BEVs due to advantageous energy storage densities, safety, infrastructure, and environmental impacts. The great potentials for increasing 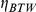 values from ethanol-ICE to the future systems (HEV and SFCV) suggest that more efficient utilization of biomass would greatly decrease greenhouse gas emissions, and biomass use could result in more benefits to the environment, rural economy, and national security than originally expected [1]. Through SFCVs, about ∼7% of annual US biomass resources may be sufficient to meet 100% of US light-duty transportation fuel needs in the future.
